# Supplementary material for: Bacillus thuringiensis Cyt Proteins as Enablers of Activity of Cry and Tpp Toxins against Aedes albopictus
Source: Toxins (Basel). 2023 Mar 10;15(3):211. doi: 10.3390/toxins15030211 (PMC10054650; doi:10.3390/toxins15030211)
Supplement: Supplementary file 1 [file toxins-15-00211-s001.zip › toxins-2243907-supplementary.pdf]

toxins

Table S1. Mortality of Aedes albopictus second instar larvae after inoculation with Cry4Aa, Cry4Ba, Cry11Aa and Cyt1Aa at high and low concentrations.

Protein	Concentration (ng/mL)	Mortality (% ± SE)
Cry4Aa	1000	90.30 ± 0.05
	100	42.84 ± 0.14
Cry4Ba	1000	100 ± 0
	100	72.51 ± 0.08
Cry11Aa	1000	73.33 ± 0.11
	100	36.67 ± 0.06
Cyt1Aa	1000	96.67 ± 0.03
	100	46.36 ± 0.05

SE: standard error.

Table S2. Raw data of the synergies between Cry and Cyt toxins.

Treatment	Concentration (ng/mL)	Mortality (% ± SE)
Cyt1Aa	30	0 ± 0
Cry4Aa	30	12.08 ± 0.03
Cyt1Aa+Cry4Aa	60	100 ± 0
Cyt1Aa	25	0 ± 0
Cry4Ba	25	17.22 ± 0.04
Cyt1Aa+Cry4Ba	50	100 ± 0
Cyt1Aa	10	0 ± 0
Cry11Aa	10	20.00 ± 0.06
Cyt1Aa+Cry11Aa	20	62.63 ± 0.06
Cyt1A-like	30	0 ± 0
Cry11Aa	30	15.19 ± 0.06
Cyt1A-like+Cry11Aa	60	85.30 ± 0.05

SE: standard error.

Table S3. Raw data of the activated Cry and Tpp toxins.

Treatment	Concentration (ng/mL)	Mortality (% ± SE)
Cyt1Aa	500	23.33 ± 0.05
Cry56A-like	500	0 ± 0
Cyt1Aa+Cry56A-like	1000	100 ± 0
Cyt1A-like	500	0 ± 0
Cry53-like	500	0 ± 0
Cyt1A-like+Cry53-like	1000	52.96 ± 0.01
Cyt1A-like	500	0 ± 0
Cry56A-like	500	0 ± 0
Cyt1A-like+Cry56A-like	1000	88.86 ± 0.01
Cyt1A-like	500	0 ± 0
Tpp36-like	500	0 ± 0
Cyt1A-like+Tpp36-like	1000	31.67 ± 0.06

SE: standard error.

Table S4. Mean lethal concentration (LC_{50}) value of BST-230.

LC	Dose (ng/mL)	Lower Limits	Upper Limits	χ^2	df	Slope	SE Slope	Intercept
LC50	39.5	26.9	53.2	2.54	4	1.50	0.212	−2.40

Treatment: Spores+crystals mixtures; χ^2: chi-square; df: degree of freedom; SE: standard error; control insects experienced no mortality in all cases.
